# Growth of High-Density Zinc Oxide Nanorods on Porous Silicon by Thermal Evaporation

**DOI:** 10.3390/ma5122817

**Published:** 2012-12-13

**Authors:** Nurul Izni Rusli, Masahiro Tanikawa, Mohamad Rusop Mahmood, Kanji Yasui, Abdul Manaf Hashim

**Affiliations:** 1 Faculty of Electrical Engineering, Universiti Teknologi Malaysia, Skudai 81310, Johor, Malaysia; E-Mail: nuruliznirusli@gmail.com; 2 School of Electrical System Engineering, Universiti Malaysia Perlis, Kuala Perlis 02000, Perlis, Malaysia; 3 Department of Electrical Engineering, Nagaoka University of Technology, Kamitomioka-machi, Nagaoka, Niigata 940-2137, Japan; E-Mails: tanikawa@stn.nagaokaut.ac.jp (M.T.); kyasui@vos.nagaokaut.ac.jp (K.Y.); 4 Faculty of Electrical Engineering, Universiti Teknologi MARA, Shah Alam 40450, Selangor, Malaysia; E-Mail: nanouitm@gmail.com; 5 Malaysia-Japan International Institute of Technology, Universiti Teknologi Malaysia, Jalan Semarak, Kuala Lumpur 54100, Malaysia

**Keywords:** zinc oxide, nanorods, porous silicon, thermal evaporation, vapor-liquid-solid

## Abstract

The formation of high-density zinc oxide (ZnO) nanorods on porous silicon (PS) substrates at growth temperatures of 600–1000 °C by a simple thermal evaporation of zinc (Zn) powder in the presence of oxygen (O_2_) gas was systematically investigated. The high-density growth of ZnO nanorods with (0002) orientation over a large area was attributed to the rough surface of PS, which provides appropriate planes to promote deposition of Zn or ZnO*_x_* seeds as nucleation sites for the subsequent growth of ZnO nanorods. The geometrical morphologies of ZnO nanorods are determined by the ZnO*_x_* seed structures, *i.e.*, cluster or layer structures. The flower-like hexagonal-faceted ZnO nanorods grown at 600 °C seem to be generated from the sparsely distributed ZnO*_x_* nanoclusters. Vertically aligned hexagonal-faceted ZnO nanorods grown at 800 °C may be inferred from the formation of dense arrays of ZnO*_x_* clusters. The formation of disordered ZnO nanorods formed at 1000 °C may due to the formation of a ZnO*_x_* seed layer. The growth mechanism involved has been described by a combination of self-catalyzed vapor-liquid-solid (VLS) and vapor-solid (VS) mechanism. The results suggest that for a more precise study on the growth of ZnO nanostructures involving the introduction of seeds, the initial seed structures must be taken into account given their significant effects.

## 1. Introduction

Zinc oxide (ZnO) with a wide direct bandgap of 3.37 eV and a large exciton binding energy of 60 meV, is one of the most extensively studied semiconducting materials for use in various types of applications [1,2,3]. One-dimensional ZnO nanostructures have become a subject of interest in the last decade due to their remarkable potential applications in electronics and optoelectronics devices such as photodetectors [2,4], light-emitting diodes [5] and solar cells [6,7,8]. Diverse group morphologies of ZnO nanostructures such as nanorods, nanowires, nanonails, nanotubes, nanocombs, nanosheets, nanobelts and nanoribbons have been synthesized by various physical and chemical fabrication techniques. These fabrication methods include thermal evaporation [3,9,10,11,12,13,14,15,16], chemical vapor deposition [17,18], hydrothermal [19], sol-gel [20,21,22] and electrodeposition [1,23]. A catalyst-free thermal evaporation method can be considered as a low cost technology for the growth of metal oxide nanostructures. A simple and economical setup makes it attractive where no expensive precursors are required. The enormous potential of this vapor-phase method is the ability to produce a variety of nanostructure morphologies with relatively high growth rate [12,24]. Moreover, if high purity metal and oxygen (O_2_) are used as the source materials and the synthesis is carried out in a properly sealed high vacuum chamber, extremely high purity and high crystallinity of ZnO nanostructures can be expected. These are the reasons why the use of a vapor-phase transport method is inevitable. However, the need of high temperature to locally heat the source materials in order to enable the vaporization might be seen as a major drawback of this method [13].

The growth of ZnO nanostructures by thermal evaporation is a bottom-up approach, and the nature of the substrate surface plays a crucial role in determining the quality of the grown nanostructures. Growth of ZnO on silicon (Si) is particularly attractive for intelligent heterogeneous integration on a Si platform. Unfortunately, it is difficult to obtain a large-area and high-density of ZnO nanostructures on the bare Si substrates because of the large mismatches in thermal expansion coefficients and lattice constants [17,25]. There are few studies reporting the feasibility of growing ZnO nanostructures on bare Si substrates at relatively low temperature by utilizing a liquid-phase solution growth method such as a hydrothermal method [26,27]. However, there are some drawbacks regarding these liquid-phase processes; (i) these methods require the introduction of ZnO seed grown by physical methods such as sputtering or spin coating prior to actual synthesis [26], hence adding more fabrication steps, (ii) this method requires a precise control of the pH and concentration of electrolytes in order to minimize the solvent inclusion into the grown materials, and (iii) the growth rate is lower than the vapor-phase process. Interestingly, by utilizing porous silicon (PS) substrate, a separate process to form the initial Zn or ZnO*_x_* seed is not needed, hence the fabrication processes are reduced to a single-step approach. Therefore, substantial efforts have been devoted to the growth of ZnO nanostructures on the PS substrates [9,10,16,25,28,29].

Many reports have thoroughly discussed the growth mechanism and explained the differences in the structures obtained regarding their experimental setup and procedures. Most studies have claimed that the growth of ZnO nanostructures on PS without a metal catalyst may be done in a vapor-solid (VS) process [9,16]. Kar and co-workers proposed a self-catalyzed vapor-liquid-solid (VLS) mechanism, which is believed to be responsible for the nucleation, and VS mechanism, to which the further longitudinal growth is ascribed [15]. However, this particular type of growth mechanism has received much less attention. In this paper, we report the formation of large-area high-density ZnO nanorods on PS substrates at growth temperatures of 600, 800 and 1000 °C by a thermal evaporation process without any catalyst. We found that the size and the geometrical morphologies of the grown nanorods are determined by the initial structures of the ZnO*_x_* seed grown at the beginning prior to the actual growth of ZnO. Based on the results, it would be more appropriate to explain the growth mechanism by a combination self-catalyzed VLS and VS mechanism. The as-grown ZnO nanorods were characterized by field-emission scanning electron microscopy (FESEM, JEOL JSM-7600 F) equipped with energy dispersive X-ray spectroscopy (EDS, Oxford Instrument, X-Max 50mm^2^), X-ray diffraction (XRD, Rigaku, RAD IIIA Cu Kα line), PL-Raman spectrometer (Horiba Jobin Yvon—A 514.5 nm line of an Ar^+^ laser at incident power of 20 mW/a 325 nm line of a continuous He-Cd laser at room temperature).

## 2. Experimental Section

### 2.1. PS Substrate Preparation

**Figure 1:**
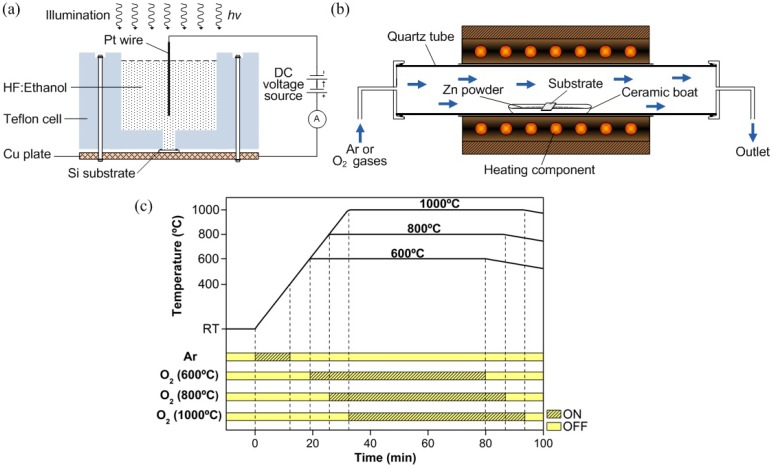
Schematic of (**a**) electrochemical etching setup; (**b**) tube furnace system and (**c**) time chart for zinc oxide (ZnO) nanorod growth by the thermal evaporation method.

The porous structure was formed on highly doped n-type Si(100) substrate by using an electrochemical etching process. Prior to the etching process, the substrate was cleaned using a standard RCA cleaning method and dried with nitrogen blow. The electrochemical etching process was performed in a Teflon cell as schematically shown in Figure 1a. The Si sample as an anode and the Pt wire as a cathode were connected to the external direct current (DC) power supply. A mixture of 48%–50% hydrofluoric (HF) acid and 95% ethanol with a volume ratio of 1:4 was used as an electrolyte. Due to the hydrophobic characteristic of the clean Si surface, ethanol was added to the aqueous HF solution to increase the wettability of the Si surface. In addition, added ethanol also helps to remove the generated hydrogen bubbles on the Si surface and hence to improve the homogeneity of the PS layer [30,31]. The process was performed at a constant current density J of 10 mA/cm^2^ for 30 min. The substrate was illuminated by halogen light (40 W) during the etching process. It has been reported that holes are required to facilitate the dissolution reactions [32,33,34]. Therefore, an external illumination was applied to realize a high concentration of holes for the etching process to take place. After the etching process, the sample was immersed in de-ionized (DI) water and dried in ambient air. The porous-structured Si substrate was characterized using FESEM to determine the pore morphology and size.

### 2.2. ZnO Nanorod Growth

High-density ZnO nanorods were synthesized in a single-zone horizontal tube furnace as schematically shown in Figure 1b. Metallic Zn powder (99.85%) and O_2_ gas (99.80%) were used as the sources without the presence of any catalyst. Prior to the growth, all of the substrates were treated with diluted HF to remove native oxides. Zn powder of approximately 0.6 g was spread evenly into a ceramic boat and the PS substrate was placed on top of the ceramic boat with the porous-structured surface facing downwards. The ceramic boat was then placed in the middle of the tube furnace. The growth temperatures were 600, 800 and 1000 °C. Ar gas of 100 sccm was flowed to remove any gas present in the quartz tube as the temperature began to rise from room temperature (RT), and the flow of Ar gas was stopped when it reached 400 °C (Zn melting point, 419 °C). The heating of Zn powder was continuously done until it reached the desired growth temperature or set point temperature (ST). At this stage, the formation of Zn or ZnO*_x_* is expected. The O_2_ gas of 200 sccm was flowed at ST for 1 h. After the growth, the furnace was turned off to cool down the sample to room temperature under natural conditions. Similar procedures were repeated for the growth on bare Si(100) substrates as comparison. The time chart of the growth process is shown in Figure 1c.

## 3. Results and Discussion

### 3.1. Morphological and Compositional Properties

**Figure 2:**
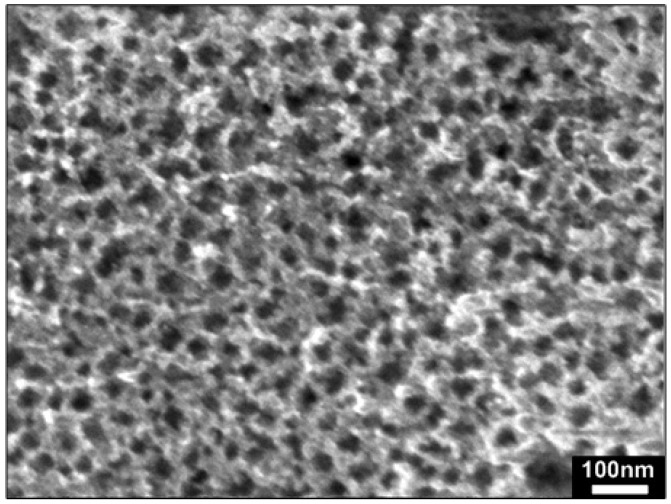
Field-emission scanning electron microscopy (FESEM) image of porous silicon (PS) substrate (top view).

**Figure 3:**
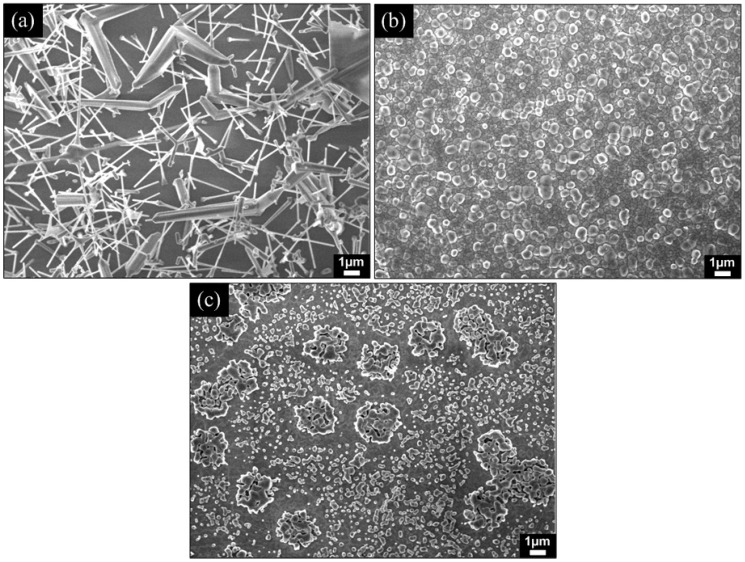
FESEM images of ZnO structures grown on Si(100) bulk substrates at different growth temperatures of (**a**) 600; (**b**) 800 and (**c**) 1000 °C.

A general morphological study indicates that the as-fabricated PS substrate possesses a high degree of porosity with a pore diameter of 15 to 40 nm as shown in Figure 2. Figure 3 shows the FESEM images of ZnO structures grown on Si(100) bulk substrates at different growth temperatures. From the images, it can be clearly observed that the growth of high-density ZnO nanorods on Si(100) bulk substrates was not achieved at all tested temperatures. The grown ZnO structures were non-uniform and sparsely distributed on the entire substrate. The nucleation of Zn and ZnO*_x_* seeds as well as the subsequent growth of ZnO nanorods on the bare Si substrate seem to be difficult with the present growth parameter due to the large lattice mismatch between ZnO and Si [35].

**Figure 4:**
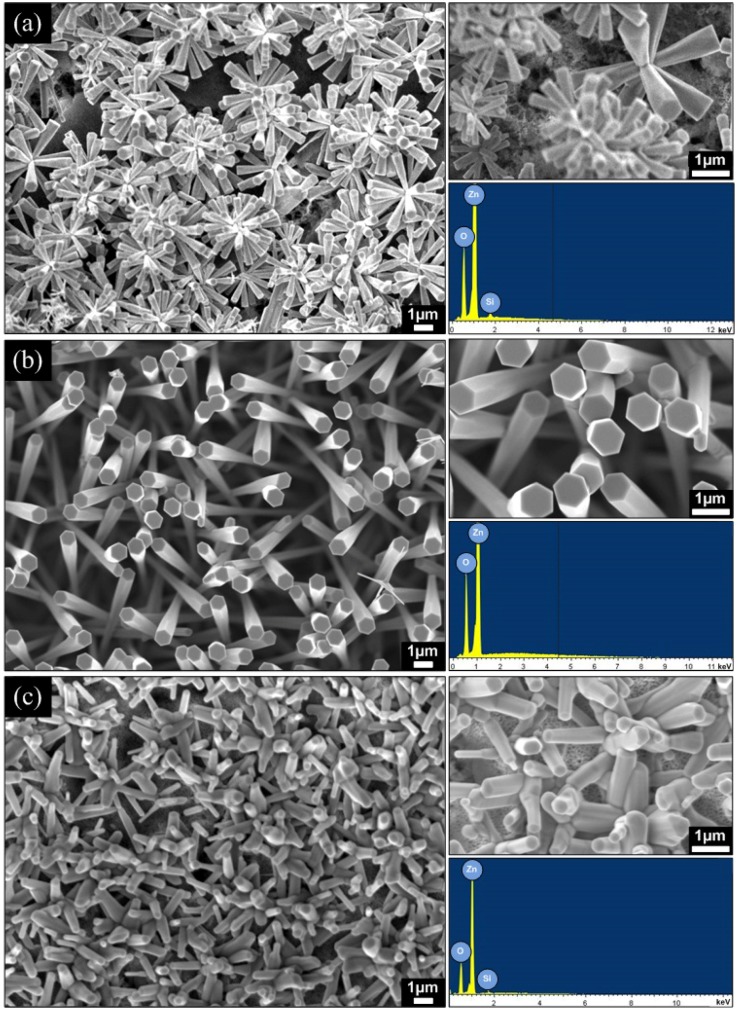
FESEM images, and energy dispersive X-ray spectroscopy (EDS) spectra of ZnO nanorods grown on PS substrates at different growth temperatures of (**a**) 600 °C; (**b**) 800 °C and (**c**) 1000 °C.

Figure 4 shows the FESEM images of ZnO nanorods grown on PS substrates at different growth temperatures. The growth of high-density ZnO nanorods was successfully obtained on the PS substrates. It is worth noting that PS has helped in providing appropriate surfaces or planes for Zn or ZnO*_x_* seed nucleation at the initial stage so that the subsequent growth of ZnO nanorods can be promoted. For example, the electrochemical etching of Si(100) may expose the (111) planes due to the high stability of (111) surfaces compared to other crystallographic planes [36,37]. These (111) planes are supposed to be the favorable sites for Zn or ZnO*_x_* species to be deposited, which may be inferred from the lower mismatch of Si(111) and ZnO(0002) planes due to the similarity in atomic arrangement [38]. This PS special structure has also been reported to have a very large internal surface that can induce large adsorption [10] of Zn or ZnO*_x_* species. As shown in Figure 4a, dense flower-shaped hexagonal faceted nanorods with a diameter range of 100–400 nm at the base side and 200–800 nm at the top side were grown at a growth temperature of 600 °C over a large area on the PS substrate. As the temperature was increased to 800 °C (Figure 4b), dense vertically aligned hexagonal faceted nanorods were grown on large area of the PS substrate. The diameters of grown nanorods were in the range of 200–500 nm at the base side and 600–950 nm at the top side. However, at a growth temperature of 1000 °C (Figure 4c), disordered nanorods with almost unchanged diameter from base side to top side in the range of 200–500 nm were grown. Their diameters were in the same range with the base diameters of nanorods grown at both 600 and 800 °C. The smaller diameter of nanorods at the base side seems to be determined by the size of ZnO*_x_* clusters. This gradual increase in diameter with the length can be explained based on the increase of Zn and O interaction as the O_2_ gas was introduced at the set point temperature. The influence of O_2_ flow rate may also contribute to such a morphology as reported by Fan *et al.* [39].

The calculated densities of nanorods for samples grown at 600, 800 and 1000 °C are estimated to be around 271 × 10^6^ cm^−2^, 41 × 10^6^ cm^−2^ and 120 × 10^6^ cm^−2^, respectively. These nanorod densities are also found to be in the same range with the other work dealing with thermal evaporation techniques [11]. However, these densities are still far smaller than the densities of nanorods or nanowires grown by the hydrothermal technique since the diameters and gap of the thermally grown nanorods are still large. Table 1 summarizes the diameter and density of the grown ZnO and in comparison with other work.

**Table 1:** Diameter and density of the grown ZnO nanorods.

	O_2_ gas flow rate (sccm)	Temperature (°C)	Diameter of nanorods (nm)	Density (cm^−2^)	Size of ZnO*_x_* seed clusters/layer (nm)
**This work**	200	600	100–400 (base) 200–800 (top)	271 × 10^6^	100–220 (cluster size)
		800	200–500 (base) 600–950 (top)	41 × 10^6^	400–840 (cluster size)
		1000	200–500	120 × 10^6^	900–1200 (layer thickness)
**[11]**	500	700	350–400 (base) 150–200 (top)	626 × 10^6^	Not reported

Elemental compositions of the as-grown ZnO nanorods on PS substrates were analyzed by EDS, and their spectra are also shown in Figure 4. Zn and O are the only detected elements (instead of the Si element in a very small atomic ratio). The ratio of Zn and O for the as-grown ZnO nanorods on PS substrates at 600, 800 and 1000 °C are found to be 0.94, 1.00 and 0.96, respectively. These high values seem to suggest that the synthesized ZnO nanorods have good stoichiometric structures. It is noted that the interaction between ZnO and PS, which lead to the formation of zinc silicate (Zn_2_SiO_4_), was not observed.

### 3.2. Crystallographic Properties

**Figure 5:**
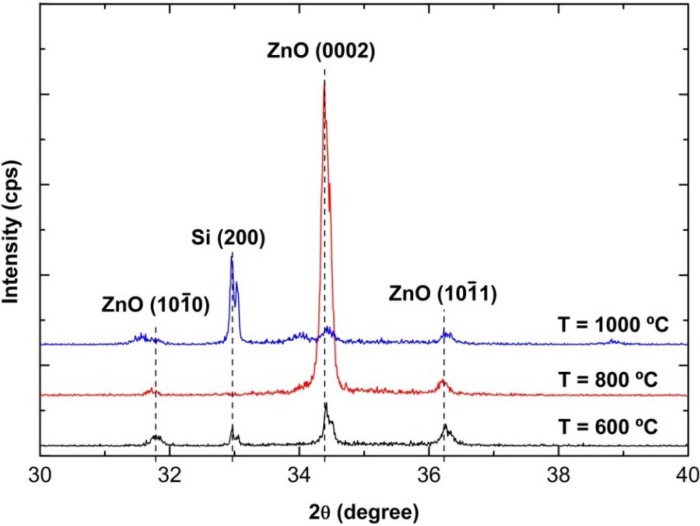
X-ray diffraction (XRD) spectra of ZnO nanorods grown on PS substrates at different growth temperatures.

Figure 5 shows XRD spectra of the as-grown ZnO nanorods on PS substrates at growth temperatures of 600, 800 and 1000 °C. The crystal planes of ($10\bar{1}0$), (0002) and ($10\bar{1}1$) belong to the hexagonal wurtzite structure [3]. A relatively high peak intensity of the (0002) plane at ~34.4° was observed for the sample grown at the temperature of 800 °C, which indicates that the preferred growth orientation of the grown ZnO nanorods is toward the *c*-axis [29], consistent with the SEM images shown in Figure 4b. However, the observed weak peak of the (0002) plane, particularly for the sample grown at 1000 °C, justified the less vertically aligned structures of nanorods toward the *c*-axis. These structures were also consistent with the SEM images (Figure 4c). Weak peaks corresponding to the ZnO ($10\bar{1}0$) and ($10\bar{1}1$) planes are observed for all samples, which are likely due to the tilted angle of the as-grown nanorods.

### 3.3. Structural Properties

**Figure 6:**
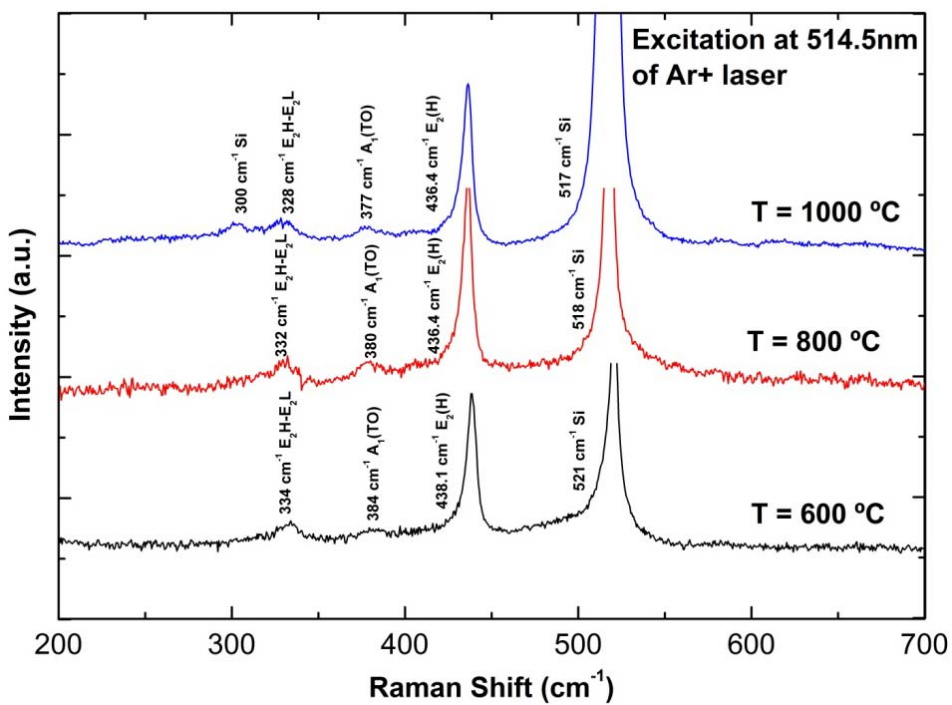
Raman scattering spectra of ZnO nanorods grown on PS substrates at different growth temperatures.

Figure 6 shows Raman scattering spectra of the grown ZnO nanorods on PS substrates at different growth temperatures. The appearance of sharp and dominant peaks at approximately 438 cm^−1^ for the sample grown at 600 °C and 436 cm^−1^ for the samples grown at 800 and 1000 °C over other peaks (except from Si substrate), corresponds to the intrinsic characteristics of the Raman-active E_2_(H) mode of wurtzite hexagonal ZnO [14,19]. The other two weak peaks; (i) at approximately 334, 332 and 328 cm^−1^ which are associated with E_2_H-E_2_L (multiple phonon process) [14,19], and (ii) at approximately 384, 380 and 377 cm^−1^, which correspond to A_1_(TO) modes [14] are also observed for the as-grown samples at growth temperatures of 600, 800 and 1000 °C, respectively. No peak attributed to E_1_(LO) mode is observed for any of the samples, thus indicating that the grown nanorods show considerably less or are free from defects such as O_2_ vacancies, Zn interstitials, or their complexes and free carriers [14,19]. A peak at ~300 cm^−1^ is observed for the sample grown at 1000 °C, which corresponds to scattering of the Si substrate [40]. The existence of a sharp and dominant peak of E_2_(H) mode without E_1_(LO) mode confirms that the grown ZnO nanorods have a hexagonal wurtzite structure with a good crystal quality.

### 3.4. Photoluminescence (PL) Properties

**Figure 7:**
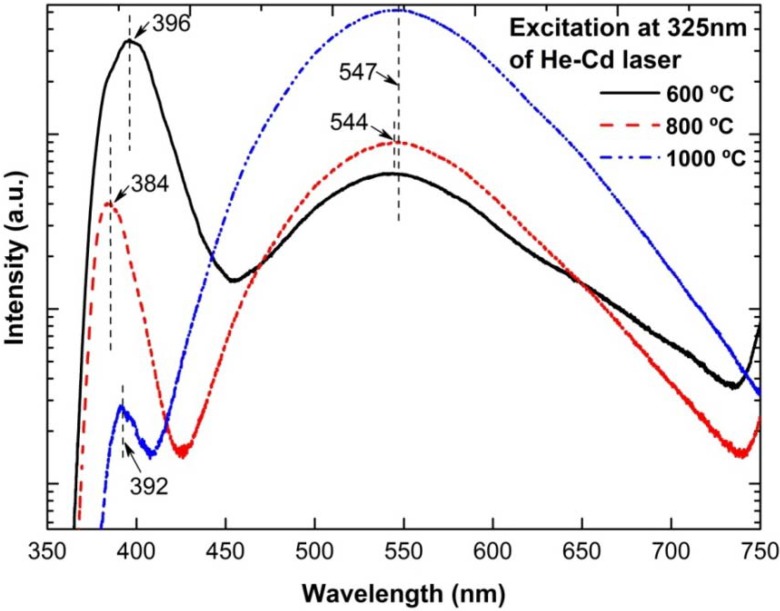
Room temperature photoluminescence (PL) spectra of ZnO nanorods grown on PS substrates at different growth temperatures.

Figure 7 shows the room temperature PL spectra for the as-grown ZnO nanorods on PS substrates, where two distinct emission bands are observed. The first band is located in the UV region at approximately 396, 384 nm and 392 nm for samples grown at 600, 800 and 1000 °C, respectively. The UV emission has been claimed to be due to the near-band edge emission (NBE) or recombinations of free excitons through an exciton-exciton collision process [3,11]. The second emission band appears in the green region of the visible spectrum at approximately 547, 544 and 547 nm for samples grown at 600, 800 and 1000 °C, respectively. This band is associated with the radial recombination of photo-generated holes with single ionized charge states of the specific defects such as O_2_ vacancies or Zn interstitials [3,11,14,19]. Although the UV emission from the sample at a growth temperature of 800 °C is weaker than the sample at 600 °C, PL blueshifts by 12 nm in the UV peak and 3 nm in the green region peak have been observed as the growth temperature is increased from 600 to 800 °C. This could be related to the increases of size and shape transitions to the well faceted hexagonal rod [41]. The strongest green emission with very weak emission in the UV region is observed as the growth temperature is further increased to 1000 °C. This tendency suggests that the growth temperature is inversely proportional to the probability to obtain high quality ZnO in this particular experimental setup at the same O_2_ rate. This result conflicts with that reported by Cheng *et al.* [12], as they found that a defect-emission-related peak was almost negligible at higher process temperature of 1000 °C, suggesting that the as-grown ZnO nanobelts were of good quality. Meng *et al.* have also reported that the grown needle-like ZnO nanowires at a low temperature of 430 °C have more O_2_ vacancies as compared to the rod-like ZnO nanowires grown at 520 °C [42]. The significant increase in the green emission intensity with the temperature suggests that there is a large fraction of O_2_ vacancies [43,44]. We speculate that these O_2_ vacancies were not from the rods but instead, remarkably, from the deposited seeds on the PS substrate. As discussed in detail in Subsection 3.5, the formed ZnO*_x_* seeds show a ratio of Zn and O composition of less than 0.76. This means that the structures are non-stoichiometric and contain high defects. Since the coverage of initial ZnO*_x_* seeds increases with the temperature from dispersely distributed nanoclusters to a thick layer, the amount of defects also increases. That is why the measured PL shows the opposite tendency. In this present study, we have only performed PL measurements for the as-grown ZnO nanorods and have not yet studied the effect of post-annealing. It is well known that the post-annealing treatment of ZnO nanorods in air or O_2_ ambient should decrease the number of O_2_ vacancies and hence improve ZnO quality. It has been reported that the post-annealing process in O_2_ remarkably improves the crystallinity of ZnO samples grown at low temperature growth techniques, *i.e.*, hydrothermal or sol-gel method [45,46]. Since the growth temperature by this thermal evaporation technique is considerably higher, the effect of the post-annealing process may not be significant. Although the ZnO*_x_* seeds are not in good crystallinity, their structures are sufficient to act as nucleation sites for the growth of high quality nanorods.

### 3.5. Possible Growth Mechanism of ZnO Nanorods

**Figure 8:**
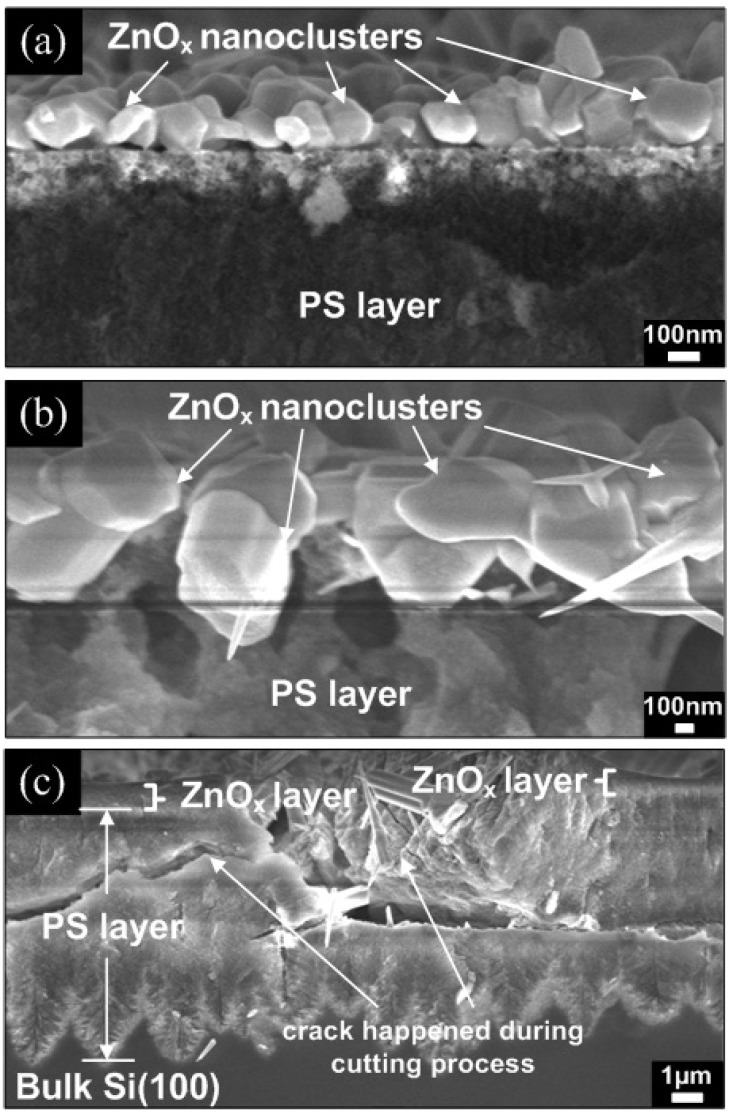
Cross sectional FESEM images of ZnO*_x_* nanoclusters and layer formed on PS substrates at (**a**) 600 °C; (**b**) 800 °C and (**c**) 1000 °C, respectively.

At first, to understand the mechanism by which the seed structure (before the introduction of O_2_ into the system) affected the geometrical morphology of subsequent ZnO nanorods, we examined the structure and compositional of the ZnO*_x_* seed using cross-sectional FESEM images and EDS. In these experiments, the furnace was immediately turned off once the temperature reached the ST. The EDS data indicate that the deposited seeds are not pure Zn, which may be inferred from the unintentionally introduced O_2_ from the ambient as the process was not performed under high vacuum conditions. Therefore, the formation of ZnO*_x_* (*x ≤* 1) compounds is observed. The ratio of Zn and O for these ZnO*_x_* are in the range of 0.25 to 0.76. We speculate that the seeds can be a mixture of both Zn and ZnO*_x_*. In the following discussion, ZnO*_x_* will be used since it is a dominant structure. As shown in Figure 8, the formation of ZnO*_x_* nanoclusters was observed at temperatures of 600 and 800 °C, while a continuous ZnO*_x_* layer was observed at a temperature of 1000 °C. The FESEM image of the ZnO*_x_* seed formed at 600 °C (Figure 8a) shows a sparse distribution of nanoclusters on the PS surface. However, denser arrays of ZnO*_x_* nanoclusters were observed at 800 °C, as shown in Figure 8b. In contrast, the ZnO*_x_* seed formed at 1000 °C exhibited a considerably thicker continuous layer, as shown in Figure 8c. The size of nanoclusters and layer thickness of ZnO*_x_* is summarized in Table 1.

**Figure 9:**
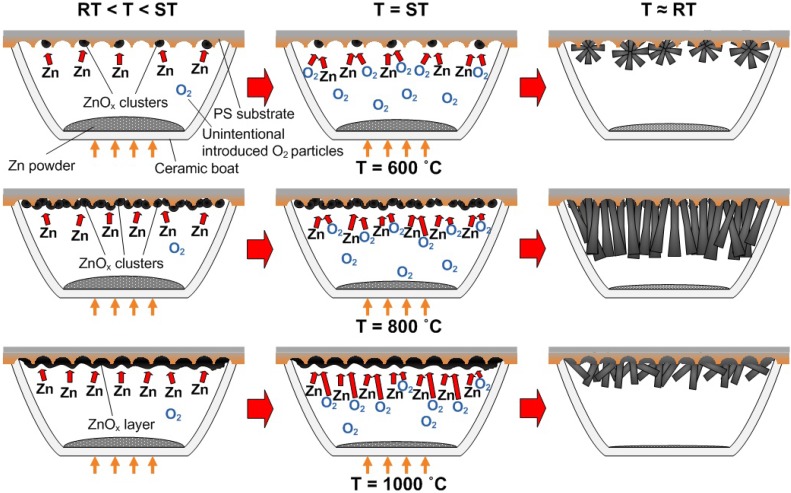
A schematic of the growth mechanism.

The proposed growth mechanism for ZnO nanorods is shown in Figure 9. We have divided the growth processes into three main stages, *i.e.*, (i) formation of ZnO_x_ clusters or layer when the furnace temperature was increased from RT to the ST, (ii) formation of additional nuclei sites on the ZnO*_x_* clusters or layer and subsequent growth of ZnO nanorods and (iii) the completion of the growth process by shutting down the furnace. The furnace was cooled down naturally to RT. In our experiments, the Zn powder source was heated continuously from 400 °C up to the ST without any supply of O_2_ or Ar gas, which is a similar approach to that reported by Srivatsa *et al.* [11]. The Zn powder begins to evaporate when exceeding the melting temperature of 419 °C. These vapors will subsequently condense as ZnO*_x_* molten liquid clusters or even as a continuous layer on the PS substrate.

At a growth temperature of 600 °C, the rate of Zn evaporation is relatively low due to the short evaporation time prior to actual growth and, therefore, might have resulted in the formation of sparsely distributed ZnO*_x_* nanoclusters. As the O_2_ gas is introduced into the system, ZnO*_x_* clusters serve as the catalyzing particles, as they will be the preferred sites for the adsorption of Zn and O_2_ atoms. Due to the continuous heating of the Zn powder source, the condensation of Zn and O vapors adds more nuclei sites surrounding the pre-formed ZnO*_x_* clusters [13]. Continuous reaction of Zn and O_2_ results in the formation of individual solid nanorods on the available nuclei sites and eventually forms flower-shaped nanorods. The growth of nanorods continues as long as the ZnO*_x_* clusters remain in the liquid state and the reactants (Zn and O) are available. The nucleation of ZnO nanorods could be ascribed to self-catalyzed VLS growth, and the subsequent longitudinal growth is attributed to the VS growth. The formation of initial nucleation was dominated by ZnO*_x_* species, therefore, the term “self-catalyzed” is used here.

At a moderately high growth temperature of 800 °C, the evaporation rate of Zn is moderately high compared to that at 600 °C due to a longer evaporation time and, therefore, results in the distribution of highly dense arrays of ZnO*_x_* clusters with increased size on the surface of PS. These ZnO*_x_* clusters also act as the preferential growth sites and enhance the adsorption of Zn and O species. Continuous condensations of Zn and O vapors have also created additional nuclei sites mostly on the top of the clusters. Thus, constant adsorption of Zn and O at the grown nuclei forms the high-density ZnO nanorods through the VLS-VS mechanism. It is noted that there is a gradual increase of nanorod diameter from the base to the top.

As the growth temperature is further increased to 1000 °C, the rate of Zn evaporation is very high and results in the deposition of a continuous layer of ZnO*_x_* on the PS substrate. This result may lead to the reduction of available nuclei sites for the growth of vertical-aligned nanorods. Consequently, disordered ZnO nanorods are grown. An increase in nanorod diameters towards the top end is not observed for this sample. This might be due to the absence of the Zn vapors near the end of the process because of the high vaporization rate of Zn powder prior to the actual growth. Strong oxidation of the source material (Zn powder) in the ceramic boat might also limit the evaporation of Zn with respect to time [11]. We believe that the size and shape of the ZnO*_x_* seeds have exerted significant influence in determining both the base diameters and the geometrical morphology of the nanorods. To achieve highly aligned, dense ZnO nanorods, the ZnO*_x_* seeds should be in the form of dense arrays of ZnO*_x_* clusters.

## 4. Conclusions

We have demonstrated the formation of high-density ZnO nanorods on PS substrates at growth temperatures of 600–1000 °C by a simple thermal evaporation of Zn powder in the presence of O_2_ gas. The high-density growth of ZnO nanorods over a large area is attributed to the rough surface of PS, which provides appropriate planes to promote deposition of Zn or ZnO*_x_* seed as nucleation sites for the subsequent growth of ZnO nanorods. It was found that the diameters and the geometrical morphologies of ZnO nanorods fabricated using this single-step method are strongly influenced by the structures of the ZnO*_x_* seeds formed during the initial growth. Dense arrays of cluster-type ZnO*_x_* seeds are better than sparsely distributed cluster-type or layer-type seeds in order to grow vertically aligned ZnO nanorods on PS substrates. The growth mechanism involved has been described by a combination of self-catalyzed VLS and VS mechanisms. The obtained results suggest that for the further precise study of the growth of ZnO nanostructures involving introduction of Zn or ZnO*_x_* seeds, the initial structures of seeds must be taken into account given their significant effects. The application of a multi-zone furnace with high vacuum capability should enable the application of the same seed structure and composition. The vertically aligned nanorods may be obtained by further optimization of seed structures and growth parameters.
